# Medium-Term Pulmonary Function Test After Thoracoscopic Lobectomy and Segmentectomy for Congenital Lung Malformation: A Comparative Study With Normal Control

**DOI:** 10.3389/fped.2021.755328

**Published:** 2021-10-27

**Authors:** Jin-Xi Huang, Song-Ming Hong, Jun-Jie Hong, Qiang Chen, Hua Cao

**Affiliations:** ^1^Department of Cardiothoracic Surgery, Fujian Branch of Shanghai Children's Medical Center, Fuzhou, China; ^2^Fujian Children's Hospital, Fuzhou, China; ^3^Fujian Maternity and Child Health Hospital, Affiliated Hospital of Fujian Medical University, Fuzhou, China; ^4^Fujian Key Laboratory of Women and Children's Critical Diseases Research, Fujian Maternity and Child Health Hospital, Fuzhou, China

**Keywords:** congenital lung malformation, segmentectomy, lobectomy, pulmonary function test, thoracoscopy

## Abstract

**Purpose:** This study aimed to compare the outcomes and pulmonary function test (PFT) of thoracoscopic segmentectomy and lobectomy in infants with congenital lung malformation and study the result of PFT on a medium-term basis.

**Methods:** The clinical data of 19 infants with congenital lung malformation who underwent thoracoscopic surgery in our hospital from January 2018 to March 2019 were retrospectively studied; these infants were paired with another 19 infants who underwent thoracoscopic lobectomy during the same period using propensity score matching. Age-matched healthy individuals with similar body sizes were recruited for PFT as the control group. Patient characteristics, postoperative PFT, and outcomes were extracted for statistical analysis.

**Results:** The average length of hospital stay did not significantly differ between segmentectomy and lobectomy groups. The segmentectomy group had more chest tube drainage than the lobectomy group. PFT 1 month after the operation showed that the tidal volume of the lobectomy group was lower than that of the segmentectomy group. Time to peak expiratory flow/time of expiration and peak flow/terminal airway velocity (V25%) indicated small airway dysfunction in the lobectomy group, and no obvious abnormalities were found in “time of inspiratory/time of expiration” in either group. Reexamination of pulmonary function 2 years after the operation showed that the small airway function of the segmentectomy group returned to normal, and no significant difference in pulmonary function was noted among the three groups.

**Conclusion:** The short-term pulmonary function recovery was better after segmentectomy than after lobectomy. Patients who underwent thoracoscopic lobectomy and segmentectomy have normal lung function 2 years after the operation.

## Introduction

Congenital lung malformation (CLM) is an uncommon pathology that involves the proliferation of terminal respiratory bronchioles at the expense of alveoli, leading to cysts of various sizes. The incidence is ~9–13.6 per 100,000 (1). It is accepted that abnormal airway patterning and branching during lung morphogenesis results in the appearance of lung cysts (2). Giubergia et al. (3) proposed that the alteration is considered a hamartomatous abnormality of the bronchial tree by some authors, whereas others favor the etiology of an arrest in the development of the fetal bronchial tree with airway obstruction.

Most infants with CLM are asymptomatic at birth, and some of them experience symptomatic infections, pneumothorax, and respiratory distress during growth. The complication rate ranges from 12 to 47% (4). Wong (5) suggested that asymptomatic CLM patients would later develop symptoms and that early surgery might be beneficial to avoid complications. Regarding the necessity of surgical treatment, Pelizzo et al. (6) considered that the potential infection and risk of malignant transformation of CLM justified surgical resection within 1 year. A radionuclide imaging study of long-term lung function in children undergoing lobectomy by Komori (7) found that the optimal age of surgery for CLM appears to be younger than 1 year to allow sufficient time for lung regrowth. With advances in surgical technology, surgeons are now able to minimize the risk of thoracoscopic surgery (8). Many pediatric thoracic diseases, including CLM, can be treated by thoracoscopic surgery (9). Thoracoscopic surgery is widely used mainly because it has the advantages of short perioperative thoracic drainage time, short hospitalization time, and fewer chest wall deformities in the long term (10). Experienced surgeons can now perform thoracoscopic lobectomy in newborns. Our center also reported thoracoscopic lobectomy for a 4-day-old neonate with a large congenital pulmonary airway malformation (11). Although thoracoscopic surgery has become a conventional technique for the treatment of CLM (12), there are few reports on segmentectomy and the clinical comparison of lobectomy and segmentectomy for CLM. We compared the outcomes and mid-term follow-up of thoracoscopic segmentectomy and lobectomy for infants and evaluated the pulmonary function of CLM patients after thoracoscopic surgery on a medium-term basis.

## Patients and Methods

The present study was approved by the ethics committee of our hospital and adhered to the tenets of the Declaration of Helsinki. The written informed consent was obtained from the parents of the children.

### Patients

From January 2018 to March 2019, we examined 19 consecutive infants who underwent thoracoscopic segmentectomy in our institution. These infants were paired with another 19 infants who underwent thoracoscopic lobectomy during the same period, based on their sex, age, weight, lesion location, and preoperative pulmonary function test (PFT), using propensity score matching. Our basic selection criteria for thoracoscopic segmentectomy was one of the following: (1) Patients with a peripheral small-sized lesion; (2) the lesion was localized to only one segment; (3) the lesions were located in two adjacent lung segments (excluding the middle lobe of the right lung). Exclusion criteria were patients with other preoperative complications such as congenital heart disease, immunocompromised state, or restrictive or obstructive chest wall disease. PFTs were carried out 1 month and 2 years after surgery.

### Surgical Technique

**Figure 1:**
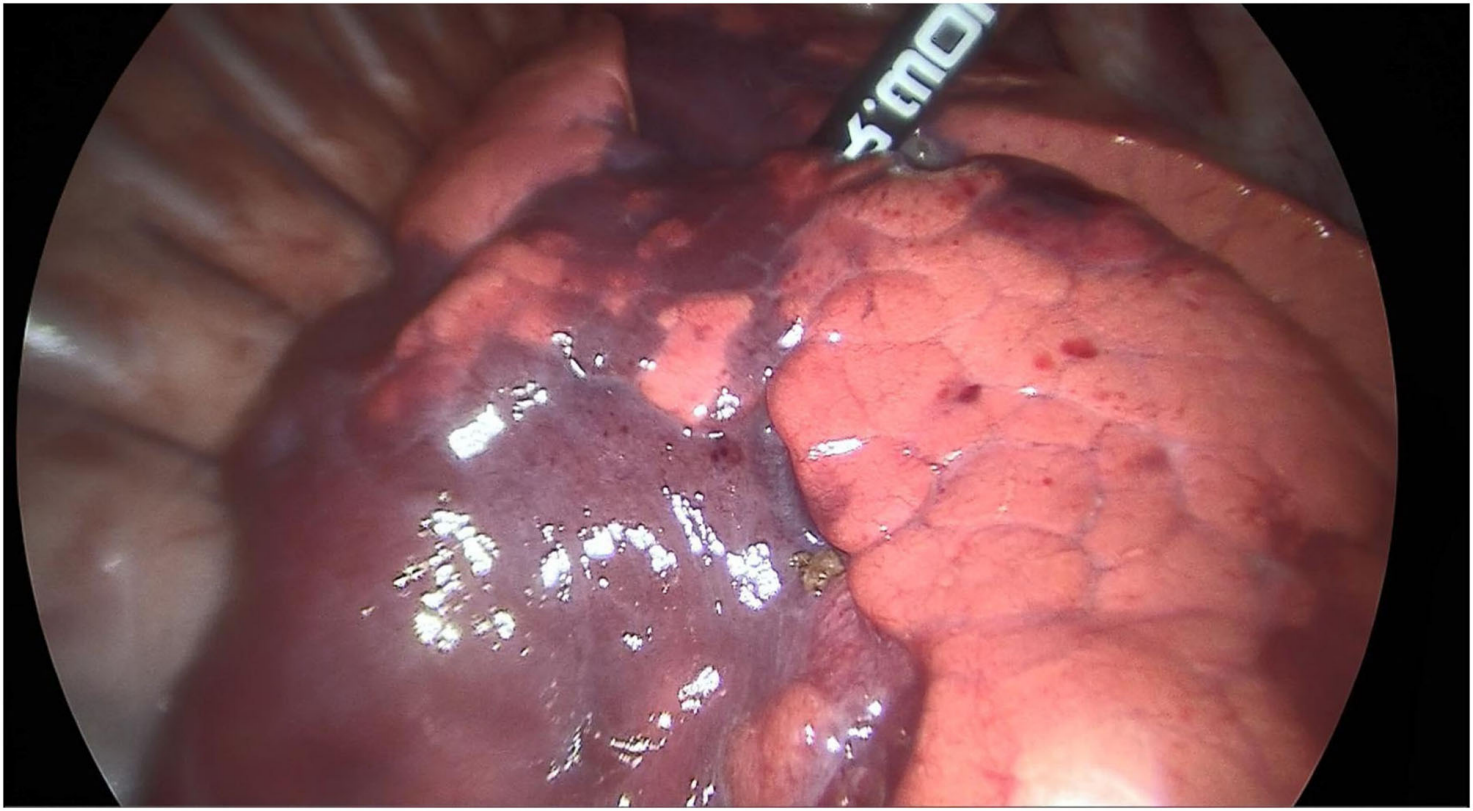
The appearance of the line between inflation and deflation under both clamping the segmental bronchi (RS9+10) and inflating the lung.

The same thoracic surgery team consecutively operated on all patients. In the segmental approach, ultrasonic scalpel and LigaSure were used to divide the vessels individually. Segmental bronchi were clamped, as inflating the operative side of the lung, the line between inflation and deflation became clear (Figure 1). The segmental bronchi were ligated with Hem-o-Lok clips (Sinolinks Clips; Sinolinks Medical Innovation, Inc. Jiangsu, China) and resected after identifying the correct segment. The lung parenchyma was dissected from the hilum to the periphery with ultrasonic scalpel and LigaSure; intersegmental veins were preserved. For lobectomy, pulmonary arteries, veins, and bronchi were dissected one by one, and the incomplete fissure was cut off with an ultrasonic scalpel and LigaSure. After reinflation of the lung and if no air leakage was detected, a closed thoracic drainage tube was indwelled, and the thoracic cavity was closed. The chest tube was removed when there was no air leak, and the amount of daily drainage was <1 ml/kg. Patients were discharged 1 day after removal of the chest tube if the follow-up chest X-ray showed no signs of pneumothorax and no signs of complications.

### Pulmonary Function Test

All patients received oral choral hydrate (0.5 ml/kg) to be kept asleep during PFTs, which were performed at least 4 h after feeding to avoid abdominal distension or vomiting. Temperature and humidity in the test room were maintained at 22°C and 40%. Infants laid flat on the testbed on their back; a face mask was attached to a face covering the nose and mouth to prevent air leakage and then connected to the MasterScreen PFT System (Jaeger, Germany). The PFTs were measured by a trained physician after smooth breathing had been established, and 15–20 cycles of tidal breathing were recorded, with five repeats. Age-matched healthy individuals with similar body sizes were recruited for PFT as the control group with informed consent obtained. Data analysis includes the following (13, 14):

① Tidal volume (VT): expressed in milliliters per kilogram.
② Tidal breath flow volume loop: The ratio of expiratory flow to inspiratory flow at 50% tidal volume (TEF50/TIF50), the ratio of the volume reaching the peak expiratory flow rate to the expiratory volume (VPEF/VE), the ratio of the time to peak expiratory flow to time of expiration (TPTEF/TE), the ratio of the peak flow to the terminal airway velocity (PF/V25%), and the ratio of the time of inspiratory to the time of expiration (TI/TE).

#### Result Criterion

① Restricted lesion: VT per kilogram is less than normal (6–10 ml/kg).
② Small airway obstruction: TPTEF/TE. The normal range is 28–55%, mild obstruction 23–28%, moderate obstruction 15–22%, and severe obstruction <15%.
③ Large airway obstruction: TI/TE. The normal range is ≥80%, mild obstruction is 60–79%, moderate obstruction is 40–59%, and severe obstruction <40%.

### Statistical Analysis

SPSS software (version 17; SPSS, Chicago, IL, USA) was used for statistical comparison between the two groups. Continuous variables were tested by Student's t-test, categorical variables were tested by the chi-square test, and one-way analysis of variance was used for statistical comparison among three groups. *P* < 0.05 was considered statistically significant.

## Results

The patient characteristics and the results of the overall operation are summarized in Table 1. The average operation time values were 86.631 ± 3.71 min in segmentectomy and 52.898 ± 0.74 min in a lobectomy. Although the volume of the chest tube in the segmentectomy group (53.162 ± 1.72 ml) was more than that in the lobectomy group (34.211 ± 0.29 ml), the length of the chest tube and the length of hospital stay were not statistically significant. Pathological examination confirmed the diagnosis of congenital pulmonary airway malformation for all cases.

**Table 1:** Patients' characteristics and outcomes.

		**Segmentectomy group**	**Lobectomy group**	***p***
Numbers		19	19	
Sex (male)		12 (63%)	12 (63%)	NS
Age (months)		4.39 ± 0.78	4.46 ± 0.91	0.790 (NS)
Body weight (kg)		7.78 ± 0.91	7.75 ± 0.88	0.900 (NS)
Location of CLM	Left	11 (57.9%)	11 (57.9%)	NS
	Right	8 (42.1%)	8 (42.1%)	NS
Duration of operation (min)		86.63 ± 13.71	52.89 ± 8.74	<0.01
Time of chest tube (days)		3.95 ± 0.85	3.74 ± 0.84	0.455 (NS)
Chest tube output (mL)		53.16 ± 21.72	34.21 ± 10.29	0.02
Postoperative Complications		3 (15.8%)	3 (15.8%)	NS
Pneumothorax		3	2	
Subcutaneous emphysema		0	1	
Length of hospital stay (days)		5.05 ± 0.78	4.79 ± 0.85	0.328 (NS)

*Results are given as the number (%), average or as the mean ± standard error of mean*.

*NS indicates not significant*.

All of the children received regular follow-up without loss. PFT was carried out 1 month and 2 years after surgery, and there was no statistically significant difference in the average weight of the children at the time of PFT. No current respiratory infections, chest wall deformity, or other factors that could impact lung function happened at the time of testing. Table 2 shows that 1 month after the operation, the lobectomy group's VT (7.6333 ± 0.523 ml/kg) was lower than that of the segmentectomy group (8.4632 ± 0.614 ml/kg) (P < 0.01). Both TPTEF/TE and PF/V25% showed that the lobectomy group had small airway dysfunction 1 month after the operation. No significant abnormalities were observed in the large airway function (TI/TE) of the two groups (0.8280 ± 0.105 in the segmentectomy group and 0.8170 ± 0.150 in the lobectomy group). Two years after the operation, PFT showed that VT was improved in the lobectomy group. A group of 19 healthy individuals, age and body size-matched, were selected as a normal control for comparison. Statistical analysis showed that the age, sex ratio, and body weight were comparable between the three groups (Table 3), and the PFTs of the segmentectomy group and lobectomy group returned to normal (not significantly different from the normal control group) (Table 4). Examinations of chest computerized tomography (CT) 2 years after the operation showed that all children recovered well without residual lesion.

**Table 2:** Pulmonary function test results (1 month after surgery).

	**Segmentectomy group**	**Lobectomy group**	***p***
VT(ml/kg)	8.463 ± 2.614	7.633 ± 3.523	<0.01
TEF50/TIF50	0.842 ± 0.120	0.831 ± 0.144	0.797 (NS)
VPEF/VE	0.260 ± 0.030	0.254 ± 0.025	0.462 (NS)
TPTEF/TE	0.319 ± 0.053	0.255 ± 0.062	<0.01
PF/V25%	1.633 ± 0.143	2.038 ± 0.754	0.027
TI/TE	0.827 ± 0.079	0.817 ± 0.107	0.731 (NS)

*Results are given as the mean ± standard error of mean*.

*NS indicates not significant*.

**Table 3:** Comparison of demographic data at PFT (2 years after surgery).

	**Segmentectomy group**	**Lobectomy group**	**Control group**	**F**	***p***
Numbers	19	19	19		NS
Sex (male)	12 (63%)	12 (63%)	12 (63%)		NS
Age (months)	28.37 ± 0.81	28.53 ± 1.09	28.11 ± 0.97	0.877	0.422 (NS)
Weight (kg)	13.67 ± 1.16	14.06 ± 1.52	14.29 ± 0.96	1.148	0.325 (NS)

*Results are given as the mean ± standard error of mean*.

*NS indicates not significant*.

**Table 4:** Comparison of pulmonary function test results in segmentectomy group, lobectomy group and control group (2 years after surgery).

	**Segmentectomy group**	**Lobectomy group**	**Control group**	**F**	***p***
VT(ml/kg)	8.051 ± 0.423	8.114 ± 0.540	8.500 ± 0.863	2.772	0.071 (NS)
TEF50/TIF50	0.831 ± 0.144	0.836 ± 0.076	0.834 ± 0.091	0.014	0.987 (NS)
VPEF/VE	0.254 ± 0.025	0.254 ± 0.027	0.244 ± 0.028	0.839	0.438 (NS)
TPTEF/TE	0.255 ± 0.062	0.271 ± 0.081	0.270 ± 0.052	0.360	0.699 (NS)
PF/V25%	1.634 ± 0.165	1.595 ± 0.316	1.676 ± 0.235	0.517	0.599 (NS)
TI/TE	0.805 ± 0.076	0.817 ± 0.159	0.844 ± 0.100	0.567	0.570 (NS)

*Results are given as the mean ± standard error of mean*.

*NS indicates not significant*.

## Discussion

The dramatic growth of minimally invasive technology had allowed segmentectomy and lobectomy to be performed thoracoscopically. In our data, the operative time in the segmentectomy group was longer than that in the lobectomy group, which was considered due to more surgical procedures. We operate in an artery-oriented manner: open the sheath of the pulmonary artery and further dissect the corresponding segmental artery, whereas lobectomy only needs to dissect the pulmonary lobar artery without dissecting the pulmonary parenchyma. It is reassuring that there was no significant difference in length of hospital stay between the two groups.

Our study found that the segmentectomy group had a greater chest tube output than the lobectomy group. Thoracic drainage after surgery was associated with lymph node dissection in adult non-small cell lung tumors (15), and thoracic drainage after thoracoscopic surgery in children was mainly derived from the exudation of intersegmental and perihilar wounds. The lung parenchyma was dissected from the lung hilum to the periphery by ultrasonic scalpel and LigaSure in the segmentectomy group, which resulted in much more intersegmental wounds and thermal damage than the lobectomy group. Another reason may be that the operation time and one-lung ventilation time of the segmentectomy group are inevitably longer than that of the lobectomy group due to the increase of operation procedure, which may lead to more pulmonary edema and pulmonary exudation (16, 17).

Pneumothorax (air-leakage >2 days) after thoracic surgery is mainly derived from the leakage of the lung parenchyma, which is related to the operation mode (18). We observed three cases of asymptomatic pneumothorax in the segmentectomy group and two cases in the lobectomy group. However, by increasing the thoracic closed drainage time, these cases were cured, and no invasive operation or secondary surgery was performed again.

Postoperative recovery of pulmonary function is another focus. The assessment of pulmonary function in children is indeed a vexing problem. The regrowth of the lung after pulmonary resection is still debated. Komori (7) tried a radionuclide CT scan and found that alveolar multiplication of the remaining lung occurs after lobectomy in patients. However, as imaging studies are difficult to quantify the extent of compensatory emphysema and increase the radiation exposure of children, they are difficult to be widely accepted by parents. Tocchioni (19) considers that the long-term prognosis after lobectomy is good for the majority of patients. For asymptomatic patients, surgery before the age of 1 year ensures optimal catch-up of pulmonary function. Lau CT's researches showed that CLM patients have normal lung function after thoracoscopic lobectomy at medium and long-term (20, 21). Those are basically consistent with our research, but there are some peculiarities to our data. In our study, 1 month after surgery, the PFT results showed that the VT of the lobectomy group was 8.4632 ± 0.614 ml/kg, which was lower than that of the segmentectomy group (7.6333 ± 0.523 ml/kg) (*P* < 0.01). In small airway functions, we found that the TPTEF/TE of the lobectomy group was 0.2550 ± 0.092 and that of the segmentectomy group was 0.3050 ± 0.060 1 month after the operation (*P* < 0.01), indicating that the lobectomy group had increased small airway dysfunction, which was also verified in the PF/V25% results. In the comparison of large airway function data, no significant differences in TEF50/TIF50 and TI/TE were noted between the two groups. We reviewed the postoperative chest radiographs of the two groups and found that the lobectomy group presented increased lung transparency and a slightly elevated diaphragmatic surface on the affected side, which was more significant than that in the segmentectomy group. We considered that the lobectomy group had more compensatory emphysema after surgery, suggesting compensatory dilation of the respiratory bronchioles and alveoli, which may have led to increased small airway dysfunction. This compensatory dilatation was still present 1 month after surgery (22). It is reassuring that we found no statistically significant differences in TPTEF/TE, PF/V25%, TEF50/TIF50, and TI/TE between the two groups 2 years after the operation. We consider that increased pulmonary parenchyma leads to improved airway function over a 2-year period of compensatory lung growth. Further prospective studies are needed to confirm the effect of short-term small airway dysfunction on the quality of life of infants.

However, there are still many limitations in our study. First, the lack of PFT data on older CLM children in our center makes it impossible to know how the compensatory growth potential changes with age. Second, obviously, the wide range of CLM is too extensive to justify segmentectomy in most cases at present. In our entire series, segmentectomy was chosen in <13% of cases. We recognized the importance of postoperative multidisciplinary follow-up, and we will also continue to observe the recovery of pulmonary function with more cases to obtain more comprehensive data and long-term follow-up outcomes.

## Conclusions

The thoracoscopic approach in infants with CLM does not interfere with normal lung function and represents a safe procedure in pediatric age. Thoracoscopic segmentectomy makes better short-term pulmonary function recovery than lobectomy. Children who underwent thoracoscopic lobectomy and segmentectomy have normal pulmonary function 2 years after the operation.

## Data Availability Statement

The raw data supporting the conclusions of this article will be made available by the authors, without undue reservation.

## Author Contributions

J-XH and HC made a significant contribution to the conception and design of the work. J-XH and S-MH drafted the work and revised it critically for important intellectual content. QC and J-XH are responsible for all aspects of the work and ensure that questions related to the accuracy or integrity of any part of the work are appropriately investigated and resolved. All authors read and approved the final manuscript.

## Conflict of Interest

The authors declare that the research was conducted in the absence of any commercial or financial relationships that could be construed as a potential conflict of interest.

## Publisher's Note

All claims expressed in this article are solely those of the authors and do not necessarily represent those of their affiliated organizations, or those of the publisher, the editors and the reviewers. Any product that may be evaluated in this article, or claim that may be made by its manufacturer, is not guaranteed or endorsed by the publisher.
