# Supplementary material for: Medium-Term Pulmonary Function Test After Thoracoscopic Lobectomy and Segmentectomy for Congenital Lung Malformation: A Comparative Study With Normal Control
Source: Front Pediatr. 2021 Oct 27;9:755328. doi: 10.3389/fped.2021.755328 (PMC8578933; doi:10.3389/fped.2021.755328)
Supplement: Supplementary file 1 [file Data_Sheet_1.PDF]

Supplementary material:

Characteristics and outcomes of 68 children underwent lobectomy from January 2018 to March 2019.

Items		Data
Numbers		68
Sex: male (%)		40 (58.8%)
Age (months)		10.67±4.01
Body weight (kg)		15.60±2.64
Location of CLM	Left	43(63.2%)
	Right	25(36.8%)
Duration of operation (min)		59.57±9.71
Time of chest tube (days)		3.84±0.90
Chest tube output (mL)		39.57±14.36
Postoperative Complications		10 (14.7%)
Pneumothorax		4
Subcutaneous emphysema		6
Length of hospital stay (days)		4.87±0.87

Results are given as the number (%) , average or as the mean ± standard error of mean.
